# Porcine circovirus type 2 in China: an update on and insights to its prevalence and control

**DOI:** 10.1186/1743-422X-11-88

**Published:** 2014-05-14

**Authors:** Shao-Lun Zhai, Sheng-Nan Chen, Zhi-Hong Xu, Man-Hua Tang, Feng-Guo Wang, Xiao-Jing Li, Bei-Bei Sun, Su-Fang Deng, Jun Hu, Dian-Hong Lv, Xiao-Hui Wen, Jie Yuan, Man-Lin Luo, Wen-Kang Wei

**Affiliations:** 1 College of Veterinary Medicine, South China Agricultural University, No. 483 Wushan Road, Tianhe District, Guangzhou 510642, China; 2 Institute of Animal Health, Guangdong Academy of Agricultural Sciences, No. 21 Baishigang Street, Wushan Road, Tianhe District, Guangzhou 510640, China; 3 Guangdong Dahuanong Animal Health Products Co., Ltd., Xinxing 527400, Yunfu, China

**Keywords:** Porcine circovirus type 2, Prevalence, Control, PCV2 vaccines, Update, China

## Abstract

Currently, porcine circovirus type 2 (PCV2) is considered the major pathogen of porcine circovirus associated-diseases (PCVAD) that causes large economic losses for the swine industry in the world annually, including China. Since the first report of PCV2 in 1998, it has been drawing tremendous attention for the government, farming enterprises, farmers, and veterinary practitioners. Chinese researchers have conducted a number of molecular epidemiological work on PCV2 by molecular approaches in the past several years, which has resulted in the identification of novel PCV2 genotypes and PCV2-like agents as well as the description of new prevalence patterns. Since late 2009, commercial PCV2 vaccines, including the subunit vaccines and inactivated vaccines, have already been used in Chinese swine farms. The aim of this review is to update the insights into the prevalence and control of PCV2 in China, which would contribute to understanding the epidemiology, control measures and design of novel vaccines for PCV2.

## Introduction

Porcine circoviruses (PCVs), the smallest known animal viruses, including PCV1 and PCV2, are members of the genus *Circovirus* in the *Circoviridae* family. PCV1 was first discovered in 1974 as a contaminant of the porcine kidney cell line PK-15 (ATCC CCL-33) and was considered non-pathogenic [1,2]; Whereas PCV2 was isolated from pigs suffering from post-weaning multisystemic wasting syndrome (PMWS) over 20 years later [3].

**Figure 1:**
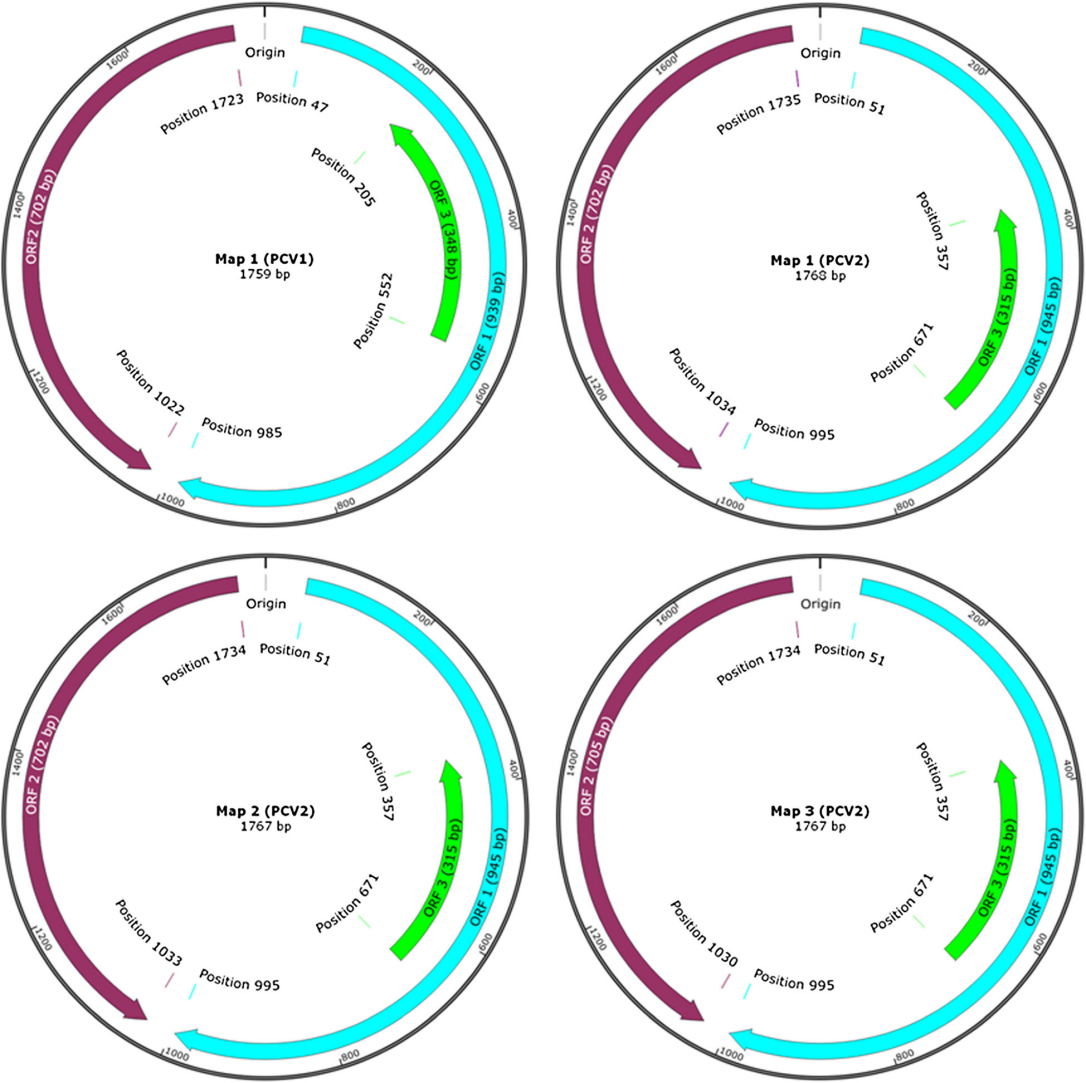
**Genome maps of PCV1 and PCV2.** Note: Generally, maps of PCV2a & PCV2e, PCV2b, PCV2d & PCV2c, are corresponding to Map 1 (PCV2), Map 2 (PCV2) and Map 3 (PCV2), respectively.

Generally, PCV1 genome consists of 1759 nucleotides (NTs), while PCV2 has 1767 or 1768 NTs. PCV1 has only one genome map (Figure 1). For PCV2, it has three kinds of genome organisation, map 1, map 2 and map 3, respectively (Figure 1). PCV2 has three major open reading frames (ORFs), ORF1 (945 NTs, position 51 to 995), ORF2 (702 or 705 NTs, position 1734/1735 to 1030/1033/1034) and ORF3 (315 NTs, position 671 to 357), respectively (Figure 1). The three ORFs encode replicase protein that involves in viral replication (to attain self-replication), the immunogenic capsid protein and the viral pathogenesis-associated protein, respectively [4-6]. One latest study reported that a newly discovered viral protein, ORF4 (180 NTs, position 386 to 565), was not essential for PCV2 replication yet having a role in suppressing caspase activity and regulating CD4(+) and CD8(+) T lymphocytes during PCV2 infection [7].

**Figure 2:**
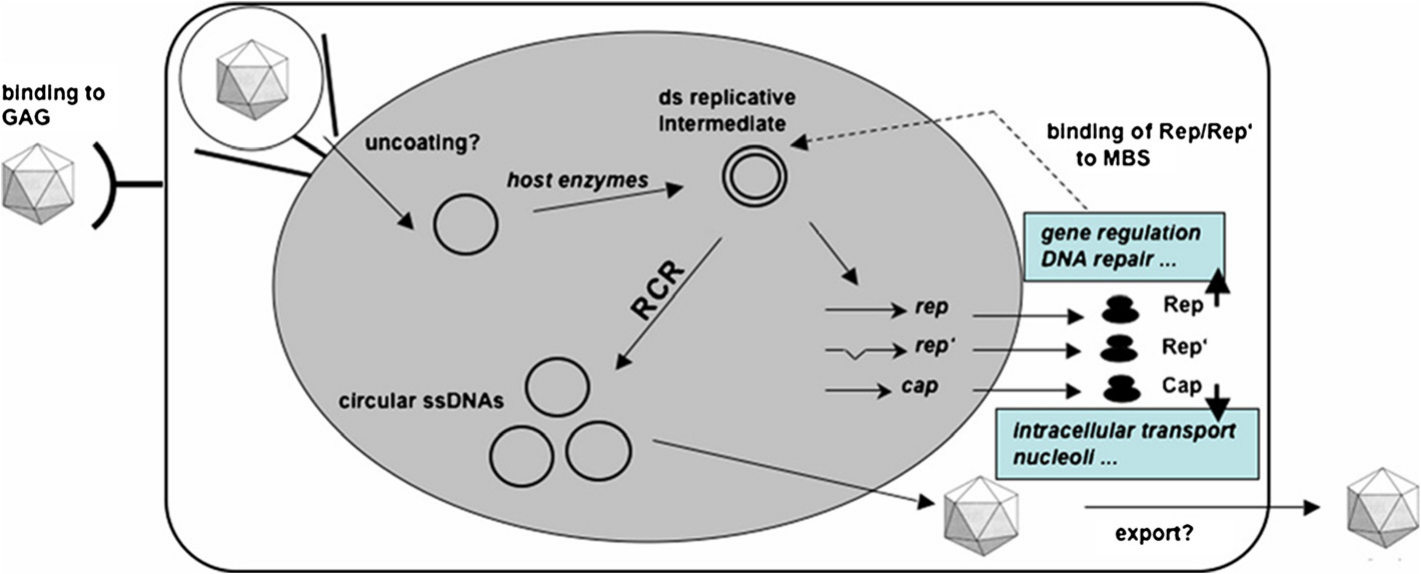
**Life cycles of PCV.** Note: The figure was showed in the previous reference [8]. Abbreviations: Glycosaminoglycans (GAG); Rolling-circle replication (RCR); Minimal binding site (MBS).

For life cycle of PCV, firstly, PCV uses glycosaminoglycans (GAG) as attachment receptors. The ssDNA genome is transported into the nucleus and converted by host enzymes into a dsDNA intermediate. The rep and cap mRNAs are transcribed to synthesize proteins, which are then imported from the cytoplasm. Rep/Rep’ then binds to the dsDNA and initiates rolling-circle replication (RCR) by introduction of a nick, which serves as primer [8]. Elongation of the primer by host enzymes leads to replication (Figure 2). Meanwhile, the Rep protein is covalently bound to the DNA and terminates the reaction by introduction of a second cleavage reaction via Tyr-93 (Figure 2). How viruses were assembled and released, have not yet been studied [8].

PCVs, especially PCV2, cause huge hazards to swine industry. At present, PCV2 is associated with a number of diseases, collectively known as porcine circovirus diseases (PCVD) in Europe [9], or porcine circovirus-associated diseases (PCVAD) in North America [10]. Apart from PMWS, PCVD/PCVAD also includes reproductive disorders [11], porcine dermatitis and nephropathy syndrome (PDNS) [12], nervous system lesions [13], porcine respiratory disease complex (PRDC) [14], enteritis [15,16], and proliferative and necrotizing pneumonia (PNP) [17,18]. Recently, a new PCV2-related disease syndrome called acute pulmonary edema (APE) that is different from the PCVD/PCVAD syndromes mentioned above was described in U.S. swine herds vaccinated with PCV2 [19].

A number of field and experimental studies that were performed in some countries of North America, Europe and Asia indicated that commercial vaccines (based on genotype PCV2a) against PCV2 were effective in many aspects, including reducing the incidence of PMWS, the number of co-infections, the severity of lesions in lymphoid tissues [20-25], the level of PCV2 viraemia and the severity of microscopically-visible lesions [26-29] as well as improving average daily weight gain and feed conversion ratios [20-25]. However, due to the subsequent possible vaccination pressure, novel variant strains or genotypes (such as PCV1/2, PCV2d) emerged in Canadian and U.S. swine herds [30-32].

In recent years, due to continuous losses caused by PCVD, the government at home and abroad supports scientists’ great deal of work on molecular epidemiology and vaccine development of PCV2. The aim of the review is to update the insights on the prevalence and control of PCV2 in China, which would help in understanding of the epidemiology, adjusting control measures, and the design of novel vaccines against PCV2.

## Review

### PCV2 Genotypes in China

**Figure 3:**
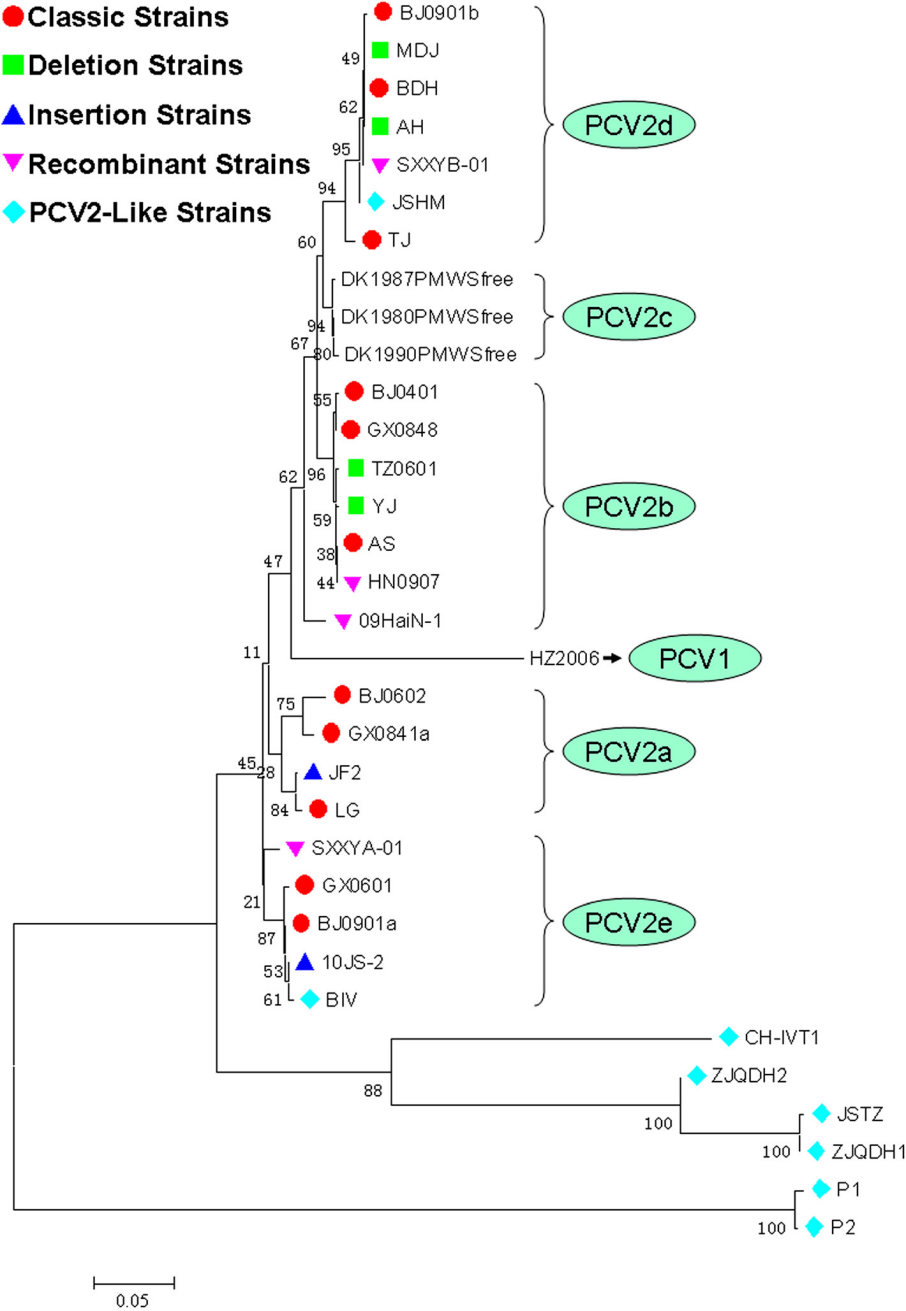
**Phylogenetic analysis of diversified PCV strains.** Note: The phylogenetic tree was constructed using the neighbor-joining method with MEGA 5.1 software. The reliability of the different phylogenetic groupings was evaluated by using the bootstrap test (1000 bootstrap replications).

After the first identification of PCV2 in China in 1998, a comprehensive molecular epidemiological survey was performed in different regions of China between 2001 and 2003 [33]. The results based on full-length ORF2 sequence level identified nine different genotypes (CHN-2A, CHN-2B, CHN-2C, CHN-2D, CHN-2E, CHN-2F, CHN-2G, CHN-2H, and CHN-2I) using restriction fragment length polymorphism (RFLP) analysis. Additional PCV2 genotypes were also reported [34-40]. Subsequently, in order to counteract the current scientific confusion on genotype nomenclature, the EU consortium on PCVD (http://www.pcvd.net/) proposed a unified nomenclature (ORF2 sequences of PCV2 are assigned into different genotypes when the genetic distance among them is at least 0.035) for the PCV2 genotype. The consortium proposed naming the three PCV2 genotypes as PCV-2a, PCV-2b, and PCV-2c [41]. On the other hand, according to the nomenclature of Wang and co-workers [42], Chinese PCV2 strains based on complete genome were classified into PCV2a, PCV2b, PCV2d, PCV2e (Figure 3), and other unidentified genotypes [42-45]. Nevertheless, Segalés et al. [46] had different comments towards the above nomenclatures and argued that PCV2 should still be divided into only three genotypes. In general, these PCV2 strains had three kinds of genome maps and two kinds of genome sizes (Figure 1) [45]. A few studies reported a number of PCV2 variant strains in China [47-50] but the method proposed by EU consortium on PCVD was not utilized. The PCV2c genotype has not been reported so far. In summary, regardless of the classification methods, the above studies demonstrated that various PCV2 genotypes did exist in swine herds of China.

### Co-Infections of Different PCV2 Genotypes in the Field in China

In 2009, Hesse et al. [51] described natural co-infections of two different PCV2 genotypes in pigs in the field for the first time. A similar molecular epidemiological survey using 118 PCV2-positive samples between 2008 and 2009 was also conducted by our group. Our result indicated that the co-infection rates of PCV2 were 32.2% (38/118) in diseased pigs and 0% (0/41) in asymptomatic pigs, respectively. Sequencing results of the 38 co-infected samples showed that the co-existent genotypes were PCV2a-PCV2b (12/38), PCV2a-PCV2d (15/38), and PCV2e-PCV2d (11/38), respectively. This was the first study demonstrating the co-existence of different PCV2 genotypes or strains in China that could help better understand the molecular epidemiology and the new infection patterns of PCV2 [52]. We believe that the presence of co-infections of different PCV2 genotypes is a more accurate description with regard to the molecular epidemiology, supported by common observations in the field.

### Co-Infections of PCV2 with Other Swine Pathogens in China

In addition to co-infections of different PCV2 genotypes, a number of studies demonstrated that PCV2 could co-infect along with by other traditional pathogens (such as porcine reproductive and respiratory syndrome virus, classical swine fever virus, porcine parvovirus, porcine pseudorabies virus, bovine viral diarrhea virus) [53-59] and emerging pathogens (porcine bocavirus, Torque teno sus virus, porcine hokovirus) [60-63].

### Novel PCV2 strains in China

#### PCV2 Strains with nucleotide deletion

**Figure 4:**
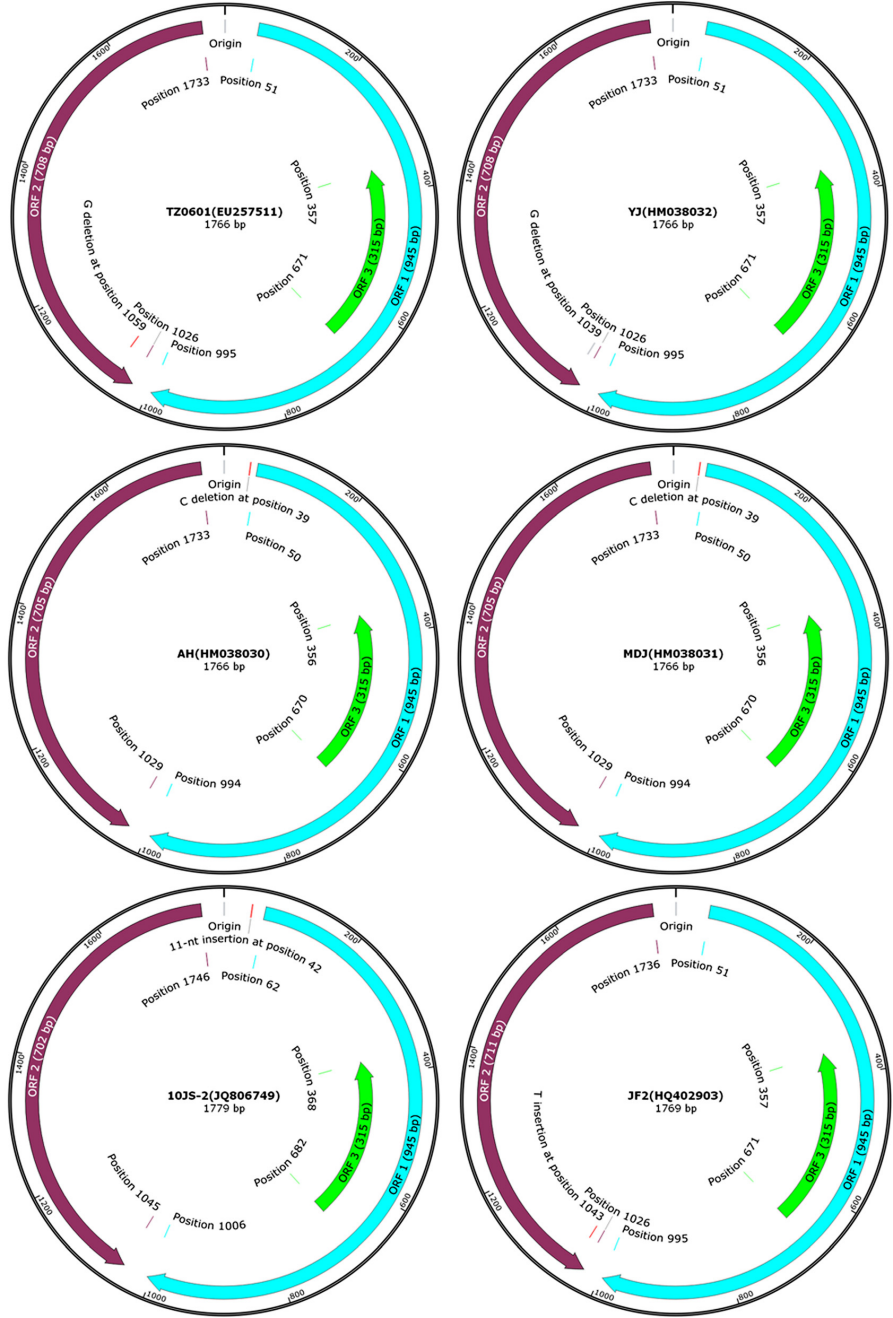
**Genome maps of four deletion strains and two insertion strains of PCV2.** For TZ0601 and YJ, the nucleotide (G) deletion occurred at position 1059 and 1039, respectively. AH and MDJ had an identical nucleotide (G) deletion at position 39. Moreover, for two insertion strains, 10JS-2 had 11-nt (AGCAGCACCTC) insertion at position 42, while JF2 had one nucleotide (T) insertion at position 1043.

In general, the full-length genome of PCV2 is 1767 bp or 1768 bp in size. However, PCV2 strains with gene deletion were identified in China. TZ0601 strain (GenBank accession number EU257511) had one nucleotide (G) deletion in the ORF2 region (Position 1733 to 1026) at position 1059 (Figure 4), resulting in C-terminal truncation of ORF2 [64]. Similarly, YJ strain (HM038032) also had one nucleotide (G) deletion in the ORF2 region (Position 1733 to 1026) (Figure 4), but the deletion occurred at position 1039 [43]. Due to one nucleotide (G) deletion in ORF2 encoding region, for the strains of TZ0601 and YJ, the size of ORF2 is lengthened from 702 bp to 708 bp. In addition, the AH strain (HM038030) and MDJ strain (HM038031) had an identical deletion (C) in the non-coding region at position 39 [43], it suggested that position 39 was a frequent deletion site, despite the fact that these deletions did not affect the ORF1 and ORF2 encoding proteins (Figure 4).

#### PCV2 Strains with nucleotide insertion

In 2004, a novel PCV2 strain, named Fh16 (AY321993), with gene insertion (11 nt, GGCAGCACCTC) at position 42 (non-coding region from position 1 to 50) was identified in non-PMWS herds in France. However, those herds presented with wasting, necrosis, and proliferous ileitis associated with Lawsonia and Pasteurella multocida. Further genetic analysis showed that Fh16 was belonged to the genotype PCV2b [35]. Furthermore, a Danish PCV2 strain DK475case (EF565360) isolated from non-PMWS herds had the identical 11-nt nucleotide insertion to the Fh16 strain [65].

PCV2 strains with nucleotide insertion also exist in China. Among them, the 10JS-2 strain (JQ806749) had 11-nt insertion (AGCAGCACCTC) at position 42 (Figure 4), which was similar to the strains Fh16 and DK475case [66]. The strain JF2 (HQ402903) had one additional nucleotide insertion (T) at position 1043 comparing to the genotype PCV2a, which led to the changes of ORF2 size (711 bp) (Figure 4) comparing to the previous strains [67]. Notably, phylogenetic analysis suggested that the strains 10JS-2 and JF2 were classified into the genotype of PCV2e and PCV2a, respectively (Figure 3).

#### PCV2 Recombinant strains

**Figure 5:**
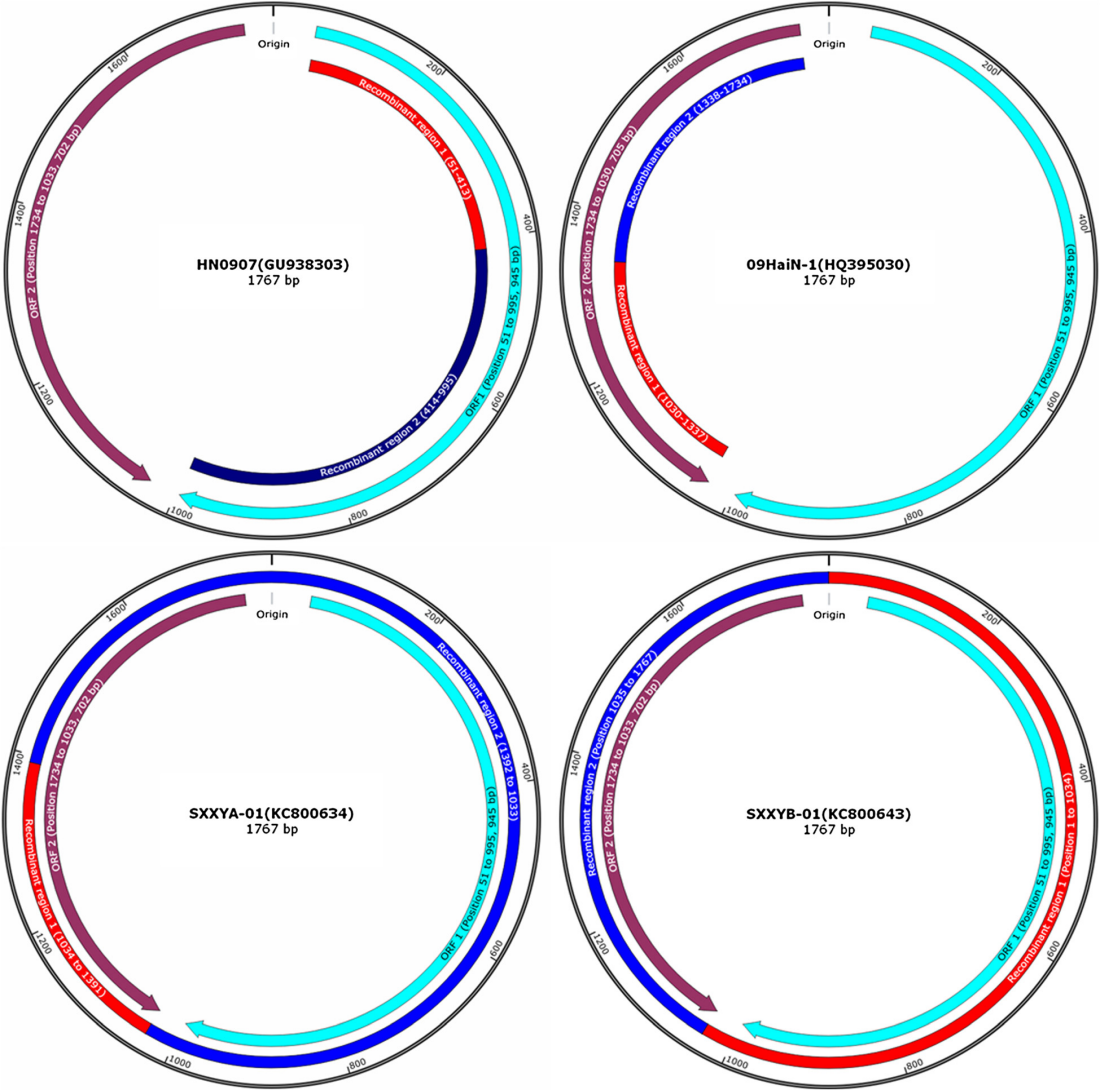
**Genome maps of four PCV2 recombinant strains.** HN0907 was an intra-genotypic recombinant between PCV2b strains, nucleotides (Position 51 to 413) were from ZhuJi2003 (AY579893, PCV2b) and nucleotides (Position 414 to 995) were from 09CQ (HQ395024, PCV2b). 09HaiN-1 was an inter-genotypic recombinant between PCV2a and PCV2b strains, it exhibited greater similarity to 336 (AY256459, PCV2a) before the breakpoint 309 of ORF2, and shared higher sequence similarity with GSLN-PCV2 (FJ948168, PCV2b) after the breakpoint 309 of ORF2. SXXYA-01 was an inter-genotypic recombinant between PCV2a and PCV2b strains, nucleotides (Position 1034 to 1391) were most from ZhuJi2003 (AY579893) and the remaining sequences were most from DTC (DQ104423). SXXYB-01 was an inter-genotypic recombinant between PCV2a and PCV2b strains, its sequence exhibited higher similarity to DTC (DQ104423, PCV2a) before the breakpoint 1034 nt and higher similarity to ZhuJi2003 (AY579893, PCV2b) after the breakpoint 1034 nt. Note: The recombinant regions were presented using red squares and blue squares.

Recombination is an important evolution pathway for viruses. PCV2 is a virus with rapid evolution. Recombination events of PCV2 have been reported from several countries including China since 2007 [30,39,51,68-75]. These recombinant strains did not only arise from intra-genotypic recombination but also inter-genotypic recombination (Figure 5). However, some recombinants’ origins might be doubtful due to artificial genome amplification and/or lack of viral plaque purification tests.

#### PCV2-Like viral agents

**Figure 6:**
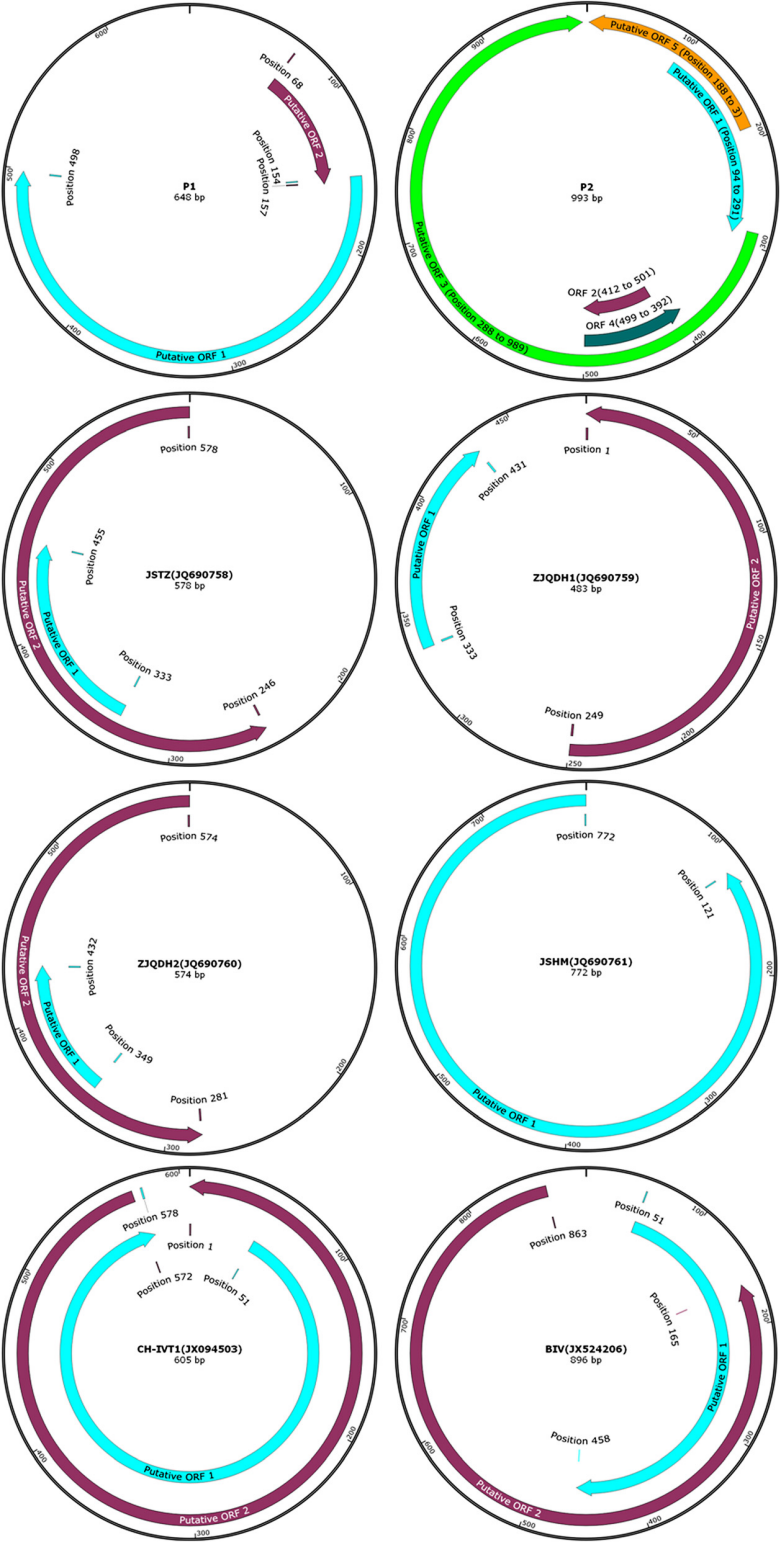
**Genome maps of eight PCV2-like agents.** For P1, twenty-two nucleotides (GGATCCACTAGTAACGGCCGCC) from 5'-terminal region might originate from porcine endogenous retroviruses and the rest of the sequence shared 98.42% identity with ORF2 of PCV2 genome (AF381175). For P2, its ORF3 sequences shared 64.7% ~ 97.4% identity with ORF2 of PCV genome. For JSTZ, ZJQDH1 and ZJQDH2, online Blastn results showed they shared hightest nucleotide identity (98% ~ 100%) with the partial sequences of PCV2 (the strain of GZ-CS1, JQ809462). For JSHM, online Blastn results showed it shared hightest nucleotide identity (98% ~ 100%) with the partial sequences of PCV2 (the strain of CQWZ12, KF742551). For CH-IVT1, online Blastn results showed it shared hightest nucleotide identity (100%) with the partial sequences of PCV2 (the strain of BF, AF381175). For BIV, online Blastn results showed it shared hightest nucleotide identity (99%) with the partial sequences of PCV2 (the strain of 10JS-2, JQ806749). Note: Complete genomes of P1 and P2 were showed in reference [78] and reference [76], respectively.

Porcine circovirus-like agents (P1 and P2) were found in China in 2008. Sequence analyses indicated that the two new agents had 648 nucleotides (P1) and 993 nucleotides (P2) in size (Figure 6), which were closely related to the isolates of PCV2 [76-78]. Epidemiology studies and animal experiments also demonstrated that P1, which caused pallor of the skin and diarrhea in pigs, had a high prevalence in Chinese swine herds [77,79]. Since then, more porcine circovirus-like strains (Figure 6) were reported [80-82]. However, these PCV2-like strains were reported only by one research team. More epidemiological work should thus be done in order to confirm their actual existence and pathogenicity.

### PCV2 DNA in Chinese commercial swine vaccines

**Figure 7:**
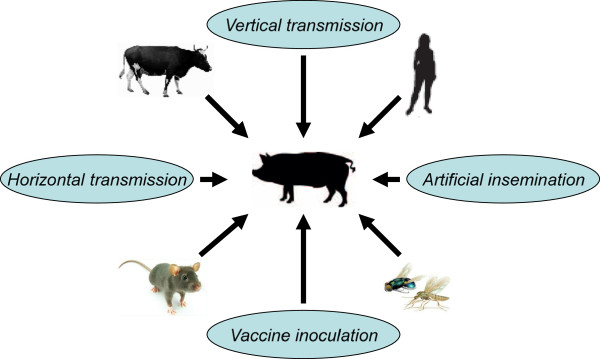
Possible infection and cross-species transmission routes of PCV in pigs.

PCV1 is non-pathogenic to pigs, and it is often detected in cell lines, pepsin, and commercial swine vaccines [83-87]. However, some studies reported that PCV2 DNA also existed in human stool, human vaccines, beef, calf bone marrow, rodents, and even commercial swine vaccines in China [88-93]. Infections of pigs and PCV-associated diseases by vaccination (Figure 7) would have occurred if PCV2 retained its infectivity in those commercial swine vaccines. To some extent, it has warned the vaccine manufacturers that good quality control measures should be taken in order to avoid the spread of PCV2 through contaminated vaccines, especially live vaccines.

### PCV2 DNA in mosquitoes collected from China

PCV2 has a high prevalence in swine herds in the world, implying that there might be other transmission routes (Figure 7). Recently, extensive PCV2 DNA was detected from water samples in Brazil, farm air in Canada, house flies in UK, and even in Culex (a kind of mosquito) (6.78%, 4/59 sampling) collected in China [94-97]. In general, mosquito is considered as an important vector for many zoonotic infectious diseases, such as Japanese encephalitis B and dengue fever. The control and killing of this vector (such as Culex and house flies) is usually overlooked under the poor swine farm conditions in China, which might exacerbate the spread of PCV2 in pig farms.

### Control of PCV2 in China

#### Vaccine

Safe and effective vaccines are considered as the best control measure for viral diseases. Commercial PCV2 vaccines (including the subunit vaccine, inactivated PCV1-2 chimera vaccine and inactivated PCV2 vaccine) have been proven to be effective in the EU, North America, and South Korea [98]. In recent years, PCV2 is given more attention by farming enterprises and the government in China. Since the first imported vaccine (Ingelvac circoflex, Boehringer Ingelheim, Ltd.) being available in China at the end of 2009, there have been at least five commercial vaccines (one subunit vaccine and four inactivated vaccines) from 16 manufacturers (Table 1). Among these vaccines, one vaccine is based on PCV2d genotype, two vaccines are based on PCV2a genotype and three vaccines are based on PCV2b genotype. In general, PCV2 vaccines induced a humoral immune response, characterized by producing neutralizing antibodies (NA) that are cross-protective against both predominant PCV2 genotypes in conventional pigs [26]. Besides, the infection tests based on SPF pigs and colostrum-deprived pigs also demonstrated that cross-protection was present between PCV2a and PCV2b [99,100]. However, one recent study reported that a PCV2 vaccine based on genotype PCV2b was more effective in protecting pigs against the effects of PCV2b than those based on the genotype PCV2a [101]. The result could act as a further support for the antigenic variability of PCV2 [64,102]. Moreover, sequence varieties between PCV2 isolates could translate to functional antigenic differences in viral neutralization in vivo [103].

**Table 1:** PCV2 vaccines used in China since 2009

**Registration Year**	**Vaccine/Strain**	**Company**	**Antigen/Genotype**	**GenBank accession No.**	**Administration**	**Licensed for**
2009	Ingelvac circoflex	Boehringer Ingelheim	ORF2 protein (PCV2a)	Not available	1 mL IM single dose	Piglets (>2 weeks of age)
2010	SH Strain	Luoyang Pulike;	Inactivated virus (PCV2d)	AY686763	1 mL IM single dose	Piglets (14–21 days);
		Nannong Hi-Tech			2 mL IM 2 doses	Gilts (45 days before mating/3 weeks later; Prenatal 30–40 days); Productive sows (Prenatal about 45 days/3 weeks later)
2010	LG Strain	Haerbin Weike;	Inactivated virus (PCV2a)	HM038034	1 mL IM single dose	Piglets (3–4 weeks);
		Shanghai Hile			2 mL IM 2 doses	Gilts (before mating/3 weeks later; Prenatal 30 days); Productive sows (Pregnancy/ Prenatal 30 days); Normal vaccination for others
2011	DBN-SX07 Strain	Fuzhou Dabeinong;	Inactivated virus (PCV2b)	HM641752	1 mL IM single dose	Piglets (14–21 days/14 days later)
		Chengdu Tecbond				
2012	WH Strain	Wuhan Keqian;	Inactivated virus (PCV2b)	FJ598044	2 mL IM single dose	Piglets (21–28 days)
		Wuhan Chopper;				
		Nangjing Tianbang;				
		Guangdong Winsun;				
		China Animal Husbandry				
2013	ZJ/C Strain	Ringpu (Baoding);	Inactivated virus (PCV2b)	Not available	2 mL IM single dose	Piglets (>14 days)
		Zhejiang Ebvac;				
		Qilu Dongbao;				
		Hangzhou Jianliang;				
		Sichuan Huapai				

#### Other measures

In addition to vaccination, other comprehensive measures have been taken in China, which mainly followed the recommendations in the developed countries, such as “Madec’s 20-point plan”, including “all-in”–“all-out” procedures, disinfection, limiting animal contact, preventing mixing of batches and cross-fostering and the isolation or euthanasia of diseased pigs; It also included the environmental factors such as the maintenance of appropriate temperature, air-flow, and space within pens; and furthermore, appropriate use of anti-parasitic treatments and vaccination [104], co-infections control [105-107], breeding and semen quality control [108-110], and herd nutrition improvement [111-113] are included as well. Nevertheless, autogenous vaccines were also considered as an effective method to prevent and control PCV2 on some pig farms from our experience.

## Conclusions

In summary, PCV2 infection is very common in Chinese swine herds. There are various PCV2 genotypes, PCV2-like strains, or mutants circulating in China. Together with the potential cross-species transmission of PCV2 (Figure 7), these factors lead to great challenges for the control of PCV2 despite the availability of vaccines. Furthermore, although cross-protection within inter-genotypes of PCV2 is present, NA only neutralizes certain PCV2 genotype but not all PCV2 genotypes [103]. At present, PCV2 infection status can be quite complex clinically, if it is not mastered well by veterinarians or practitioners. It might hinder choosing the right choice of PCV2 vaccines and thus not conducive to the prevention of PCV2. To overcome this problem, it is necessary to perform the detection and sequencing of PCV2 antigen. In addition to effective comprehensive measures, the development of polyvalent vaccines (such as PCV2a + PCV2b + PCV2d) or universal vaccines against all PCV2 genotypes are needed in the future.

## Competing interests

The authors declare that there is no competing interests regarding the publication of this article.

## Authors’ contribution

SLZ structured the review and prepared the draft of the manuscript. SNC collected the literatures and prepared the manuscript. BBS, SFD and JH prepared the tables and figures. ZHX, MHT, FGW, XJL, DHL, XHW, JY, WKW and MLL revised the manuscript. All authors read and approved the final manuscript.
